# Anti-β_2_GPI/β_2_GPI complex promotes thrombosis by activating the P2Y_2_/MAPKs pathway to increase human neutrophil peptides

**DOI:** 10.1371/journal.pone.0322447

**Published:** 2025-05-22

**Authors:** Xin Guan, Wen Liu, Tianfeng Gao, Wenying Jin, Yueqiu Gao, Huiyuan Tan, Lujie Guo, Yanfen Zhang

**Affiliations:** Department of Laboratory Diagnosis, The Second Affiliated Hospital of Harbin Medical University, Harbin, Hei Longjiang Province, China; Yale University, UNITED STATES OF AMERICA

## Abstract

Anti-β_2_ glycoprotein I (Anti-β_2_GPI) antibodies are a heterogeneous group of antiphospholipid antibodies targeting β_2_ glycoprotein I (β_2_GPI). High titer of anti-β_2_GPI antibodies is a risk factor for thrombosis in antiphospholipid syndrome (APS). Although it has been shown that anti-β_2_GPI antibodies can induce neutrophil activation involved in thrombosis, the underlying mechanism remains unclear. In this study, we analyzed the clinical data of thrombotic patients who were positive or negative for anti-β_2_GPI antibodies, as well as healthy individuals. The results showed that the percentage and absolute count of neutrophils, serum levels of human neutrophil peptides (HNPs), and HNP mRNA levels were significantly higher in the anti-β_2_GPI-positive patient group compared to the healthy control group. Notably, when compared to the anti-β_2_GPI-negative patient group with similar neutrophil percentages and counts, the serum HNPs levels were also significantly elevated in the anti-β_2_GPI-positive patient group. In vitro, we further showed that anti-β_2_GPI and β_2_GPⅠ complex (anti-β_2_GPI/ β_2_GPⅠ complex) induced a concentration - and time-dependent increase in HNPs, which was mediated through P2Y_2_ receptors on the surface of neutrophils. Meanwhile, we found that intracellular signaling pathways P38MAPK (P38 mitogen-activated protein kinase) and ERK (extracellular signal-regulated kinase) were also involved in the generation of HNPs. We also found that high levels of human neutrophil peptide-1 (HNP-1) could induce the production of procoagulant factors von Willebrand factor (vWF) and P-selectin in endothelial cells through the nuclear factor-κB (NF-κB) signaling pathway, which increased the risk of thrombosis.

## Introduction

Antiphospholipid syndrome (APS) is an autoimmune disease characterized by the presence of antiphospholipid antibodies. This antibodies can manifest a variety of clinical phenotypes, including arterial/venous thrombosis and obstetric complications, of which arterial/venous thrombosis is the main pathophysiological hallmark [1]. Some population-based studies have reported an estimated prevalence of 6–9% in patients with obstetric onset and 9–10% in patients with arterial events and venous thromboembolism, and antiphospholipid antibodies are the leading cause of inducing thrombosis in APS [2].

β_2_ glycoprotein I (β_2_GPI) is the primary antigenic target of antiphospholipid antibodies in APS. The binding of β_2_GPI to anionic phospholipids on the plasma membrane will cause conformational changes of β_2_GPI and expose hidden epitopes in D1. Anti-β_2_ glycoprotein I antibodies (anti-β_2_GPI antibodies) targeting D1 are closely related to thrombosis [3]. It has been reported that complexes formed by anti-β_2_GPI and β_2_GPⅠ can bind to anionic phospholipid complexes expressed on the surface of platelets, endothelial cells, and monocytes to promote thrombosis [4–6]. As important cells in innate immunity, neutrophils also participate in inflammatory response and promote thrombosis in APS patients [7–8]. However, the mechanism of anti-β_2_GPI and β_2_GPI complex on neutrophils involved in thrombosis has not been clearly studied.

Human neutrophil peptides (HNPs) are cysteine-rich cationic peptides, a member of α-defensins [9]. The expression of HNPs is the highest in neutrophils and mainly exists in the aniline blue granules. HNPs contain many subtypes, among which human neutrophil peptide1–3 (HNP1–3) is the most abundant neutrophil granule protein, and the three only differ in amino acids at the N-terminal. At the same time, human neutrophil peptide-1 (HNP-1) accounts for the highest proportion, accounting for about 70% [10]. HNPs are closely related to the progression of various inflammatory diseases and are a relevant indicator for evaluating the severity of associated diseases [11–12]. However, with the development of research, plasma HNP1–3 concentration is related to the incidence of myocardial infarction and cardiovascular disease mortality [13–15]. The endothelium is the sole interface between blood and tissues, and local endothelial cells possess antithrombotic properties. Upon activation by external stimuli, endothelial cells release a series of procoagulant substances, such as P-selectin and von Willebrand factor (vWF), which can induce a hypercoagulable state in the blood and may even directly mediate thrombus formation [16–17]. Previous studies have demonstrated that high levels of HNPs can induce endothelial cell dysfunction [18–19], but the effects of HNPs on vWF and P-selectin are unknown.

The formation mechanism of HNPs varies according to different cell types. In activated neutrophils, adenosine triphosphate (ATP) acts on the P2Y_2_ receptor on the membrane surface of its own neutrophils to promote neutrophil migration, degranulation, and immune defense [20–21]. Meanwhile, it has been shown that in bronchial asthma, the expression of α-defensins in neutrophils can be altered by intervening on P2Y_2_ receptors [22]. Therefore, we propose that the anti-β_2_GPⅠ/β_2_GPⅠ complex may induce an increase in the release of HNPs from neutrophils through the P2Y_2_ receptor.

In this study, we aimed to investigate the mechanism by which the interaction between anti-β_2_GPI/β_2_GPI complex and the P2Y_2_ receptor on neutrophils induces an increase in HNPs. Additionally, we examined the effects of high levels of HNP-1 on the expression and release of vWF and P-selectin in endothelial cells in vitro. This study may provide a new theoretical basis for the involvement of anti-β_2_GPI and β_2_GPⅠ complex in inflammatory thrombosis.

## Materials and methods

### Reagents and antibodies

HNP1–3 ELISA kit, vWF ELISA kit were purchased from Jianglai Biological (Shanghai, China). Human peripheral blood neutrophil isolation kit, ATP, Sulforaphane, PDTC inhibitor were purchased from Solarbio Science and Technology Beijing, China). Anti-p38MAPK phosphorylation antibody, anti-ERK phosphorylation antibody, anti-P-selectin antibody, anti-NF-κB P65 antibody, anti-NF-κB P65 phosphorylated antibody were purchased from Immunoway. Anti-vWF antibody was purchased from Affbiotech. Anti-β-actin antibody, goat anti-mouse secondary antibody, goat anti-rabbit secondary antibody were purchased from Zhongsugi Jinqiao Biotechnology (Beijing, China). Mouse normal immunoglobulin (M-IgG), Bovine Serum albumin (BSA), SB203580 inhibitor, PD98059 inhibitor were purchased from Biyuntian Biotechnology (Beijing, China). Purified anti-β_2_GPI antibody, β_2_ glycoprotein were purchased from Sino Biological Company(Beijing, China). Purified HNP-1 was purchased MCE Company(Shanghai, China).

### Study subjects

From September 26 to November 16, 2023, 30 individuals with anti-β_2_GPI levels over 20 RU/ml, diagnosed with thrombosis or a history of thromboembolism confirmed by CT, MRI and other imaging examinations in the Second Affiliated Hospital of Harbin Medical University were selected as the anti-β_2_GPI-positive patient group (Among them, 20 patients were diagnosed with cerebral infarction, 4 with pulmonary embolism and 6 with coronary artery embolism). At the same time, 30 individuals with anti-β_2_GPI level less than 20 RU/ml who were diagnosed with thrombosis or had a history of thromboembolism confirmed by CT, MRI and other imaging examinations were selected as the anti-β_2_GPI-negative patient group (Among them, 20 patients were diagnosed with cerebral infarction, 4 with pulmonary embolism and 6 with coronary artery embolism). Thirty individuals who underwent routine physical examination during the same period were randomly selected as the healthy control group. Exclusion criteria: combined with serious underlying diseases such as liver and kidney; Blood system diseases; Malignant tumors; Additional thrombotic risk factors and recent (within the past 2 weeks) use of anticoagulants, including medications such as aspirin and clopidogrel; Long-term smoking; Elevated BMI. At the same time, clinical data of all subjects from September 26 to November 16, 2023 were retrospectively collected. This study was reviewed and approved by the ethics committee of the Second Affiliated Hospital of Harbin Medical University, and all subjects signed informed consent.

### Isolation of neutrophils from peripheral blood

Neutrophils were extracted from human peripheral blood by adding 4 ml of solution A, 2 ml of solution C, and peripheral blood sequentially to a 15 ml centrifuge tube and centrifuging for 30 min at 1200 g at 25°C. After centrifugation, the neutrophils between solution A and solution C were carefully aspirated into new centrifuge tubes. Erythrocyte lysate was added to lysate the cells twice, washed once with phosphate buffer solution (PBS), and then the neutrophils were resuspended in RPMI 1640 medium. Trypan blue staining is commonly used to evaluate the survival rate of neutrophils. Meanwhile, the purity of neutrophils was evaluated by Giemsa staining.

### Cell culture

Human umbilical vein endothelial cells (HUVEC) were purchased from Rosetta Stone Biotechnology Co., LTD.(Taiyuan, Shanxi). HUVEC were cultured in DMEM medium containing 10% FBS and 1% streptozotocin in a cell incubator with 5% CO_2_ and temperature maintained at 37°C. The passage times of HUVEC used in the experiment were 3–5 passage number. The confluency of the cells used in the experiment was 50%-60%.

### ELISA

The collected serum samples from the clinical anti-β_2_GPI positive patient group, anti-β_2_GPI negative patient group, and healthy control group were directly added to the detection wells of the ELISA kit, with a sample volume of 100 μl per well. Neutrophils were inoculated into the six-well plate at a density of 5 × 10^6^/ml, and each stimulation group was stimulated for 1 hour and 4 hours. HUVEC were inoculated into the twelve-well plate at a density of 1 × 10^6^/ml, and each stimulation group was stimulated for 1 hour and 4 hours. After the incubation periods, the culture supernatants from each group were collected and added to the detection wells of the ELISA kit, with a sample volume of 100 μl per well. The plates were then incubated at 37°C for 1 hour. According to the instructions of the kit, add 100 μl of biotin antibody, incubate at 37°C for 1 hour, wash the plate for 3 times, add 100 μl of enzyme conjugate working solution, incubate at 37°C for 30 minutes, wash the plate for 5 times, add 90 μl of substrate, incubate at 37°C for 15 minutes, and finally, add 50 μl of termination solution, detect the OD values of the wells at 450 nm, and plot the standard curve at standard level to calculate the HNP1–3, vWF level.

### Real-time reverse transcription PCR (RT-PCR) analysis

The extracted neutrophils were added with 1 ml Trizol reagent, left at room temperature for 1 hour, then added with 100 μl chloroform substitute, mixed thoroughly on a vortex oscillator, left at room temperature for 2 minutes, centrifuged at 12000g for 15 minutes at 4°C, then sucked the supernatant, added with 500 μl isopropanol, left at room temperature for 10 minutes. The supernatant was discarded by centrifugation at 12000g for 10 min at 4°C. The total RNA level in the precipitate was measured by a UV spectrophotometer. Trans Criptor First Strand cDNA Synthesis Kit Reverse transcription for cDNA synthesis. HNP1–3 and P2Y_2_ primers were added, and 2^-∆∆Ct^ was calculated from the Ct value using β-actin as an internal reference. The primer sequences used were as follows (5 ‘-3’):

P2Y_2_ forward: GCTACAGGTGCCGCTTCAAC;

reverse: AGACACAGCCAGGTGGAACAT;

HNP1–3 forward: AGGAGAACGTCGCTATGGAA;

reverse: TCCCTGTAGCTCTCAAAGCA;

β-actin forward: GGGAAATCGTGCGTGACATTAAG;

reverse: TGTGTTGGCGTACAGGTCTTTG

### Western blotting analysis

Neutrophils and HUVEC were fully lysed in RIPA lysis suspension (lysate: phenylmethyl sulfonyl fluoride (PMSF): phosphatase inhibitor volume ratio: 100:1:2) on ice, and the total protein was extracted by centrifuge. The total protein level was detected by BCA method, 10% separation gel was configured, and then sodium dodecyl sulfate polyacrylamide gel electrophoresis was performed (SDS-PAGE). Rabbit anti-human P-P38MAPK, P-ERK, NF-κB P65, and P-NF-κB P65 were added, mouse anti-human β-actin primary antibody was incubated at 4°C overnight, the membrane was washed, horseradish peroxidase labeled goat anti-rabbit and goat anti-mouse secondary antibodies were added, and the membrane was incubated at 37°C for 1 hour. Enhanced chemiluminescence (ECL) imaging.

### Immunofluorescence

HUVEC were seeded in a 12-well plate at a density of 1 × 10^6^/ml and incubated in a CO_2_ incubator for 4 hours. After cell attachment, the culture medium was changed to each stimulation group. After stimulation, the culture medium was discarded, fixed with 4% paraformaldehyde for 15 minutes, and the supernatant was discarded, and permeabilized with 0.1% Triton X-100 for 10 minutes at room temperature. The supernatant was discarded, washed thrice in PBS and blocked with 5% BSA for 2 hours. After blocking, rabbit anti-human vWF, P-selectin, and NF-κB primary antibodies were added and incubated at 4°C overnight, washed 5 times with PBS, added secondary antibodies, and incubated at room temperature in the dark for 1 hour. DAPI was added to reverse transfection to block the slides, and the images were observed under fluorescence microscope.

### Cell proliferation assay

HUVEC were seeded in 96-well plates (1 × 10^4^ cells per well), at least three multiple Wells in each group, and cultured overnight. After cell adherence, the culture medium was discarded, the negative control group was replaced with a new culture medium, the stimulation group was replaced with 5 μg/ml and 10 μg/ml HNP-1 culture medium, and the cells were cultured in a CO_2_ incubator. After 24, 48, and 72 hours, each well was replaced with a medium containing CCK-8 working solution, and after continued incubation in a CO_2_ incubator for 2 hours, the absorbance was measured at 450 nm wavelength by a multifunctional microplate reader.

### Statistical analysis

Normally distributed data are presented as mean$\overset{―}{x}$± SD, while non-normally distributed data are expressed as median (interquartile range, IQR), specifically M (P25, P75). Comparisons between two groups were performed using independent samples t-tests. For comparisons among multiple groups, data were first analyzed by one-way analysis of variance (ANOVA). Statistical analyses and graphical representations were conducted using GraphPad Prism software (version 8.0.2, GraphPad Software, La Jolla, CA, USA). When P < 0.05, it was considered statistically significant.

## Results

### Clinical data analysis

In this study, a total of 90 clinical specimens were collected, including 30 thrombotic patients positive for anti-β_2_GPI antibodies, 30 thrombotic patients negative for anti-β_2_GPI antibodies, and 30 healthy controls. As shown in Table 1, the mean age of the anti-β_2_GPI-positive patient group was (57.6 ± 15.65) years, that of the anti-β_2_GPI-negative patient group was (54.0 ± 14.63) years, and that of the healthy control group was (49.8 ± 16.05) years.

**Table 1:** Demographic data of the enrolled subjects.

Demographic data	Anti-β_2_GPI positive patient group (n = 30)	Healthy control group (n = 30)	Anti-β_2_GPI negative patient group (n = 30)
Age (range)	57.6 ± 15.65	49.8 ± 16.05	54.0 ± 14.63
Gender(Male/female)	30(13/17)	30(12/18)	30(13/17)
Number of neutrophils (×10^9^/L)	4.74(3.62,6.27)	3.64(3.18,4.55)	5.40(3.57,6.22)
Neutrophil number percentage (%)	62.70(58.45,75.97)	59.25(54.25,63.65)	66.45(61.02,74.90)

**Fig 1:**
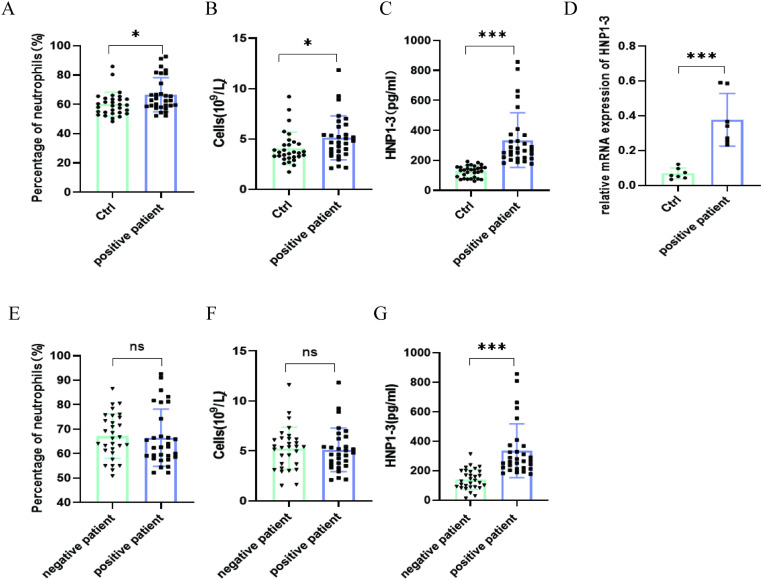
Analysis of clinical data of thrombosis patients and healthy subjects (A and B). Compared with healthy controls, the percentage of neutrophils and the number of neutrophils were increased in the anti-β_2_GPI-positive patient group. **(C)**. ELISA showed that the serum level of HNP1-3 in anti-β_2_GPI-positive patient group was higher than that in the healthy control group. **(D)**. The level of HNP1-3 mRNA in anti-β_2_GPI-positive patient group was higher than that in the healthy control group. **(E and F)**. The neutrophil percentage and neutrophil count were similar between the anti-β_2_GPI-positive patient group and those anti-β_2_GPI-negative patient group. **(G)**. Compared to the anti-β_2_GPI-negative patient group, the serum levels of HNP1-3 were elevated in the anti-β_2_GPI-positive patient group. NS means not significant *P < 0.05. **P < 0.01; ***P < 0.001.

There was no statistically significant difference in demographic age between the anti-β_2_GPI-positive patient group and the healthy control group (P > 0.05), ensuring comparability between groups. Compared to the healthy control group, the anti-β_2_GPI-positive patient group exhibited increased percentages and absolute counts of neutrophils (Fig 1A and 1B). Further analysis of serum samples revealed that the serum levels of HNP1–3 in the anti-β_2_GPI-positive patient group were significantly higher than those in the healthy control group (Fig 1C). Seven random samples from each group were selected, and peripheral blood neutrophils were extracted to compare HNP1–3 mRNA levels. The HNP1–3 mRNA levels in peripheral blood neutrophils from the anti-β_2_GPI-positive patient group were higher than those in the healthy control group (Fig 1D). To further investigate whether elevated HNP1–3 levels are associated with the activation of neutrophils by high levels of anti-β_2_GPI/β_2_GPI complex in vivo, we compared the anti-β_2_GPI-positive patient group with the anti-β_2_GPI-negative patient group, which had similar neutrophil percentages and counts (Fig 1E and 1F). There was no statistically significant difference in demographic age between these two groups (P > 0.05), ensuring their comparability. Study results indicated that serum HNP1–3 levels were higher in the anti-β_2_GPI-positive patient group compared to the anti-β_2_GPI-negative patient group (Fig 1G).

### Results of viability and purity identification of neutrophils

**Fig 2:**
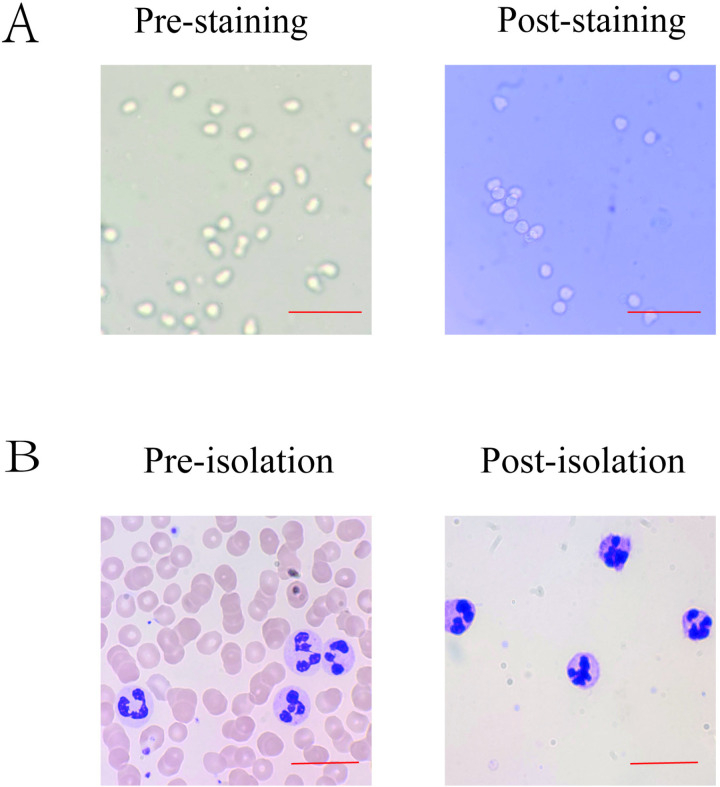
Results of viability and purity identification of neutrophils (A). Representative microscopy images of isolated neutrophil viability as assessed by trypan blue exclusion assay. Ruler:50μm **(B)**. Representative microscopy images of isolated neutrophil purity as assessed by Giemsa staining solution. Ruler:20μm.

Neutrophils were isolated from peripheral blood using a human neutrophil isolation kit. The viability of the neutrophils was assessed using Trypan blue exclusion, and the results demonstrated that the viability exceeded 95% (Fig 2A). The purity of the neutrophils was evaluated using Giemsa staining, which indicated that the purity was greater than 98% (Fig 2B). These findings confirm that the isolated neutrophils exhibit high viability and purity, making them suitable for subsequent studies.

### Anti-β_2_GPI/β_2_GPI-induced HNP1–3 release increased and was dependent on P2Y_2_ receptor activation

**Fig 3:**
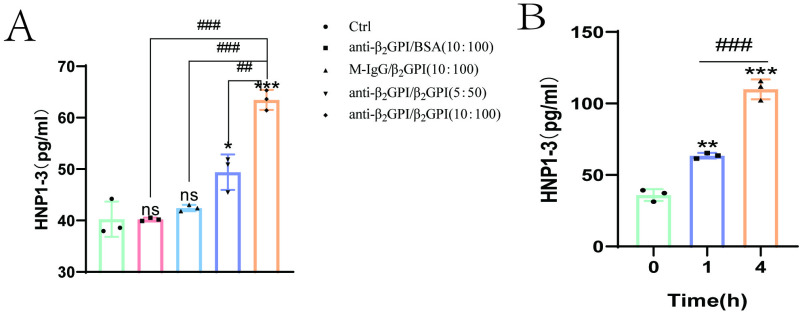
Anti-β_2_GPI/β_2_GPI stimulated increased release of HNP1-3 from neutrophils (A). Neutrophils were incubated with anti-β_2_GPI/β_2_GPI (binding ratio 1:10) for 1 hour, HNP1-3 was increased in a concentration-dependent manner. The combination of anti-β_2_GPI (10 μg/ml) and β_2_GPI (100 μg/ml) was significantly more effective in enhancing the release of HNP1-3 compared to the individual components alone. [control group, anti-β_2_GPI (10 μg/ml)/BSA (100 μg/ml) group, M-IgG (10 μg/ml)/β_2_GPI (100 μg/ml) group, anti-β_2_GPI(5 μg/ml)/β_2_GPⅠ (50 μg/ml) group, anti-β_2_GPI (10 μg/ml)/β_2_GPⅠ (100 μg/ml) group]. **(B)**. Anti-β_2_GPI(10 μg/ml)/β_2_GPⅠ (100 μg/ml) stimulated HNP1-3 release in a time-dependent manner (0, 1, 4 **h)**. Compared with the control group, *P < 0.05; **P < 0.01; ***P < 0.001, compared with anti-β_2_GPI/β_2_GPI complex incubation group, ^#^P < 0.05; ^##^P < 0.01; ^###^P < 0.001.

To determine the effect of anti-β_2_GPI/β_2_GPⅠ on the release of HNP1–3 from neutrophils, We treated neutrophils with different concentrations of anti-β_2_GPI and β_2_GPⅠ complex [anti-β_2_GPI (5 μg/ml)/β_2_GPⅠ (50 μg/ml), anti-β_2_GPI (10 μg/ml)/β_2_GPⅠ (100 μg/ml)] for different times (0, 1, 4 hours). We used 1:10 as the effective ratio of anti-β_2_GPI to β_2_GPⅠ binding [23], M-IgG and BSA were used as isotype controls. The results demonstrated that the anti-β_2_GPI/β_2_GPI complex more effectively induced an increase in HNP1–3 levels compared to anti-β_2_GPI or β_2_GPI alone. Moreover, the anti-β_2_GPI/β_2_GPI complex induced an increase in HNP1–3 release in a concentration- and time-dependent manner (Fig 3A and 3B). Further experiments were performed with anti-β_2_GPI (10 μg/ml)/β_2_GPⅠ (100 μg/ml) stimulation for 1 hour.

**Fig 4:**
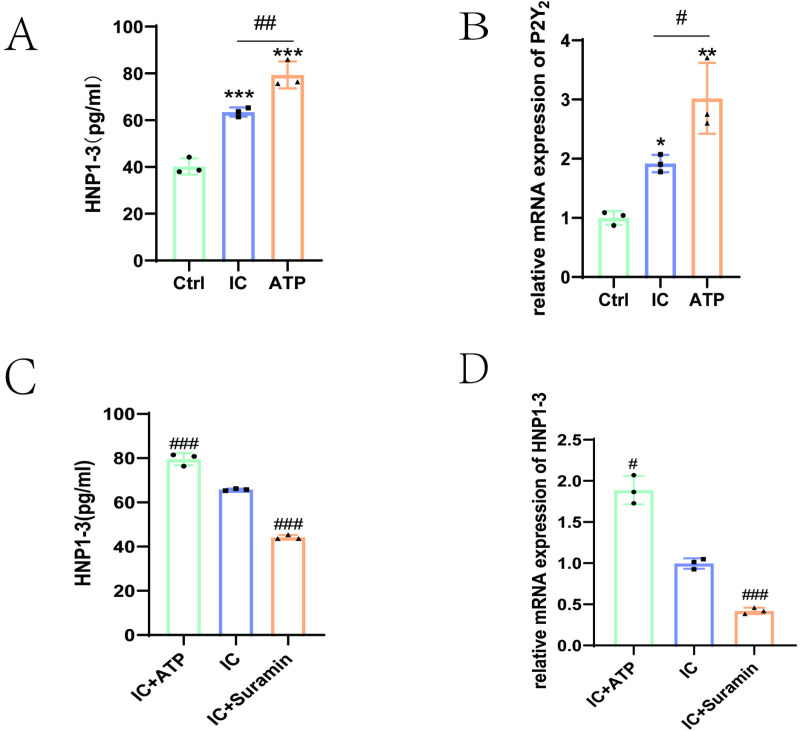
Anti-β_2_GPI/β_2_GPI stimulated HNP1-3 release from neutrophils was dependent on P2Y_2_ receptor (A). Incubation of neutrophils with anti-β_2_GPIβ_2_GPⅠ complex (IC), ATP (200 μM) significantly increased HNP1-3 release as compared with the control group. **(B)**. RT-PCR showed that the expression of P2Y_2_ receptor mRNA was increased in neutrophils cultured with anti-β_2_GPI/β_2_GPⅠ complex (IC). **(C)**.ELISA showed that ATP (200 μM) enhanced HNP1-3 release from neutrophils cultured with anti-β_2_GPI/β_2_GPⅠ complex (IC). However, suramin (100 μM) abolished the release of HNP1-3 by anti-β_2_GPI/β_2_GPⅠ complex (IC). **(D)**.The expression of HNP1-3 mRNA measured by RT-PCR was consistent with that by ELISA. Compared with the control group, *P < 0.05; **P < 0.01; ***P < 0.001, compared with anti-β_2_GPI/β_2_GPI complex (IC) incubation group, ^#^P < 0.05; ^##^P < 0.01; ^###^P < 0.001.

Here, we investigated whether the anti-β_2_GPI/β_2_GPⅠ (IC) involved in HNP1–3 release through the action of P2Y_2_ recepors and ATP, a specific activator of the P2Y_2_ receptor, significantly increased the release of HNP1–3 from neutrophils (Fig 4A). Meanwhile, anti-β_2_GP/β_2_GPⅠ significantly promoted P2Y_2_ receptor mRNA levels compared with the control group (Fig 4B). To further investigate the effect of P2Y_2_ receptors on HNP1–3 release, we treated neutrophils with receptor inhibitor suramin and activator ATP. The activator ATP (200μM) significantly enhanced HNP1–3 release induced by anti-β_2_GPI/β_2_GPⅠ. The release of HNP1–3 induced by anti-β_2_GPI/β_2_GPⅠ was abolished by inhibitor suramin (100 μM). At the same time, RT-PCR results showed that the ATP + IC group significantly increased the mRNA level of HNP1–3, and the suramin + IC group significantly decreased the mRNA level of HNP1–3. Its results are consistent with the ELISA results described above (Fig 4C and 4D).

### Effect of anti-β_2_GPI/β_2_GPⅠ on the release of HNP1–3 through P2Y_2_ receptor regulation of phosphorylation of P38MAPK and ERK1/2 signaling pathways

**Fig 5:**
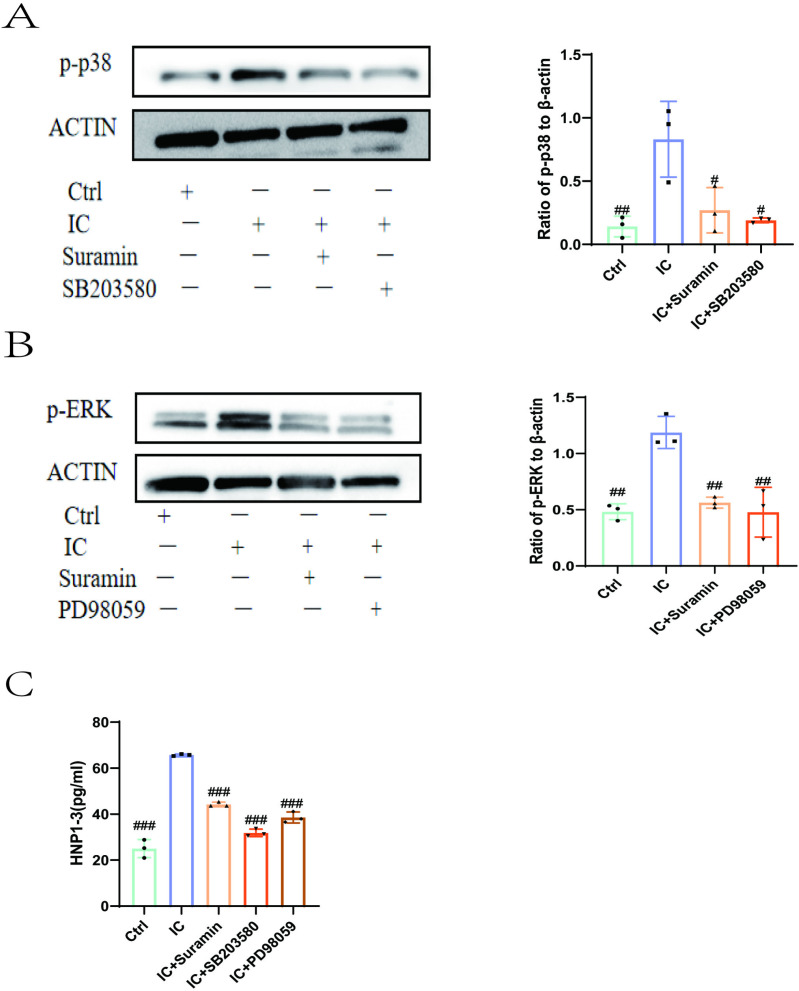
Anti-β_2_GPI/β_2_GPI regulate phosphorylation of P38MAPK and ERK1/2 signaling pathways through P2Y_2_ receptors (A and B). Western blotting show Neutrophils incubated with anti-β_2_GPI/β_2_GPⅠ complex (IC) promoted the phosphorylation of P38MAPK and ERK1/2. The phosphorylation of P38MAPK and ERK1/2 induced by anti-β_2_GPI/β_2_GPⅠ complex (IC) was abolished by specific inhibitors suramin (100 μM), SB203580 (10 μM) and PD98059 (50 μM). **(C)**. Specific inhibitors suramin (100 μM)、SB203580 (10 μM) and PD98059 (50 μM) abolished HNP1-3 release induced by anti-β_2_GPI/β_2_GPⅠ complex (IC). Compared with anti-β_2_GPI/β_2_GPI complex (IC) incubation group, ^#^P < 0.05; ^##^P < 0.01; ^###^P < 0.001.

ERK1/2 and p38MAPK-mediated cascades play an important role in the production of inflammatory cytokines. At the same time, studies have shown that the two signaling pathways are also involved in the process of neutrophil oxidative burst and degranulation. Here, we pretreated neutrophils with 100 μM suramin (P2Y_2_ receptor inhibitor), 10 μM SB203580 (P38 MAPK inhibitor), and 50 μM PD98059 (ERK1/2 inhibitor) for 30 minutes and then stimulated them with anti-β_2_GPI/β_2_GPI for 30 minutes. Western blotting showed that the anti-β_2_GPI/β_2_GPⅠ promoted the phosphorylation of P38 MAPK and ERK1/2. Meanwhile, suramin (P2Y_2_ receptor inhibitor), SB203580 (P38 MAPK inhibitor), and PD98059 (ERK1/2 inhibitor) significantly inhibited the phosphorylation induced by anti-β_2_GPI/β_2_GPⅠ and the release of HNP1–3 (Fig 5). Studies have shown that the anti-β_2_GPI/β_2_GPⅠ regulates the phosphorylation of P38MAPK and ERK1/2 signaling pathways through P2Y_2_ receptors to promote the release of HNP1–3.

### HNP-1 inhibits HUVEC proliferation and induces vWF and P-selectin production

**Fig 6:**
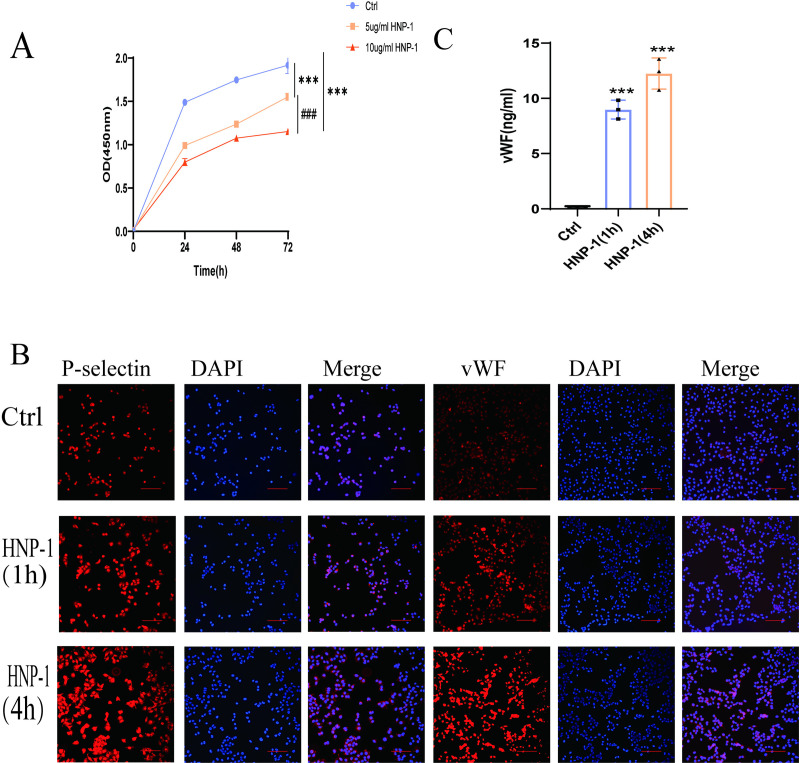
HNP-1 inhibited the proliferation of HUVEC and produced vWF and P-selectin increase (A). HNP-1 (5μg/ml, 10μg/ml) inhibited the proliferation of HUVEC in a concentration dependent manner. **(B)**.Immunofluorescence showed that HUVEC were incubated with HNP-1 (5 μg/ml), and HNP-1 (5 μg/ml) can promote the intracellular expression of P-selection and vWF in HUVEC. It is time dependent. The red fluorescence represented the immunoreactivity of P-selectin and vWF, while the blue color confirmed the presence of cells stained with DAPI reagent. Ruler: 100μm. **(C)**. The level of vWF in the supernatant of HUVEC incubated with HNP-1 (5 μg/ml) also increased with time. Compared with the control group, *P < 0.05; **P < 0.01; ***P < 0.001, compared with HNP-1 incubation group, ^#^P < 0.05; ^##^P < 0.01; ^###^P < 0.001.

We treated endothelial cells with high levels of HNP-1, and the effect of HNP-1 on proliferation inhibition of HUVEC was gradually significant with increasing time (Fig 6A). Our study found that compared to the control group, treatment of HUVEC with 5 μg/ml HNP-1 for 0, 1, and 4 hours significantly upregulated the intracellular expression of vWF and P-selectin, as shown in Fig 6B. Additionally, ELISA measurements of vWF levels in the cell supernatant revealed that HNP-1 promotes the release of vWF from HUVEC in a time-dependent manner (Fig 6C).

### The induction of vWF and P-selectin by HNP-1 from HUVEC was dependent on the activation of NF-κB signaling pathway

**Fig 7:**
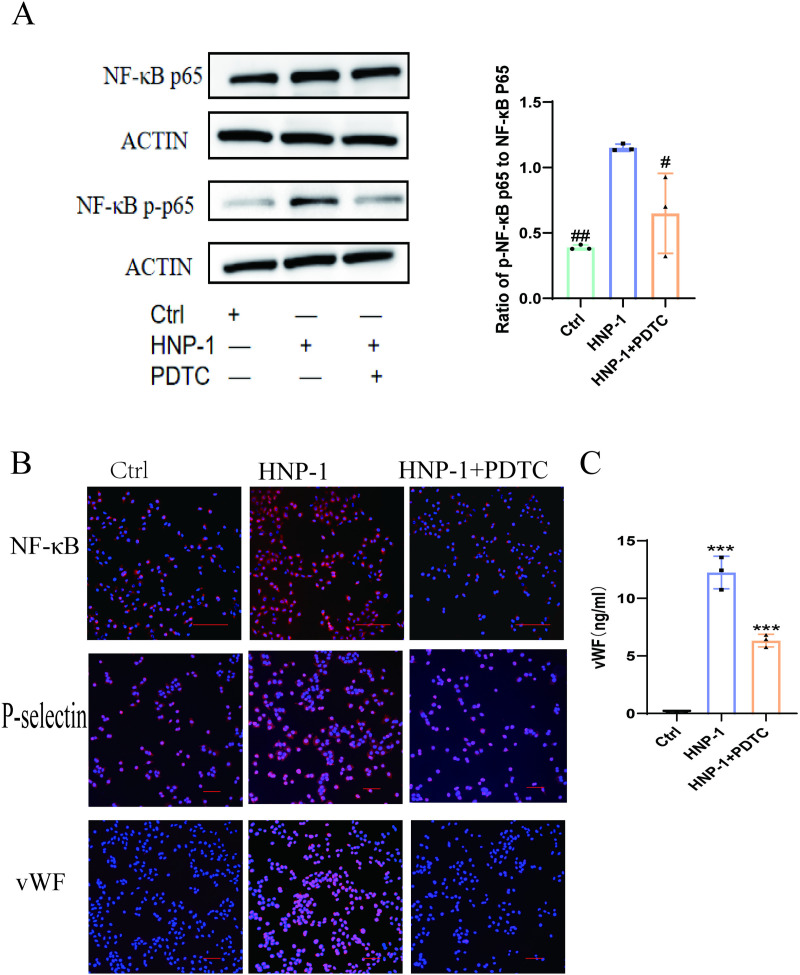
The induction of vWF and P-selectin from HUVEC by HNP-1 was dependent on the activation of NF-κB signaling pathway (A). Western blotting showed that incubation of HUVEC with HNP-1 (5 μg/ml) significantly promoted the phosphorylation of NF-κB. HNP-1-induced phosphorylation of NF-κB was abolished by treatment with the specific inhibitor PDTC (5 μg/ml)). **(B)**. Immunofluorescence showed that HNP-1 (5 μg/ml)) significantly promoted the nuclear translocation of NF-κB. The specific inhibitor PDTC (5 μg/ml)) abolished HNP-1 induced NF-κB nuclear import and induction of P-selectin and vWF in HUVEC. Red fluorescence represents the immunoreactivity of NF-κB, P-selectin and vWF. While blue confirmed the presence of cells stained with DAPI reagent. Ruler: 100μm. **(C)**.ELISA showed the level of cell supernatant, inhibitor PDTC abolished the release of vWF by HNP-1 from HUVEC. Compared with the control group, *P < 0.05; **P < 0.01; ***P < 0.001, compared with HNP-1 incubation group, ^#^P < 0.05; ^##^P < 0.01; ^###^P < 0.001.

In the HNP-1 + PDTC group, endothelial cells were first treated with 5 μg/ml PDTC (NF-κB inhibitor) for 1 hour, followed by co-culture with 5 μg/ml HNP-1 for 3 hours. Western blotting analysis revealed that compared to the control and HNP-1 + PDTC groups, the HNP-1 group significantly promoted the phosphorylation of NF-κB p65 (Fig 7A). Immunofluorescence results showed that in the control group, NF-κB p65 in HUVEC was primarily localized in the cytoplasm, indicating a resting state. In contrast, the HNP-1 group significantly facilitated the nuclear translocation of NF-κB p65, indicating activation of the NF-κB pathway. In the HNP-1 + PDTC group, PDTC abolished the HNP-1-induced nuclear translocation of NF-κB p65 and the expression of intracellular vWF and P-selectin (Fig 7B). This finding is consistent with our measurements of vWF levels in the cell supernatant (Fig 7C).

## Discussion

Anti-β_2_GPI antibody is a member of the antiphospholipid antibody family, and its complex with β_2_GPI is the primary pathogenic mechanism that induces thrombosis in APS [24]. However, more and more studies have found that the binding of anti-β_2_GPI/β_2_GPI complexes to anionic phospholipid complexes expressed on the cell surface leads to cellular activation and a shift towards a pro-inflammatory and pro-thrombotic phenotype, constituting the first hit in the induction of APS-related thrombosis. Simultaneously, factors such as infections and inflammation induce cellular dysfunction, serving as the second hit that ultimately results in thrombus formation [25]. As important cells in inflammatory response mediators, neutrophils have been paid more and more attention to the mechanism of immune thrombosis induced by vascular inflammation [26]. However, the mechanisms by which the anti-β_2_GPI/β_2_GPI complex interacts with neutrophils to contribute to thrombosis have not been fully elucidated. It is currently believed that the anti-β_2_GPI/β_2_GPI complex may promote coagulation through the activation of neutrophils, leading to the release of a series of inflammatory mediators such as neutrophil extracellular traps (NETs), proteases, and granule proteins [27–28]. HNPs are cationic peptides with broad-spectrum antimicrobial activity, mainly found within the azurophilic granules of neutrophils. When pathogens invade the body, neutrophils are activated to degranulate and release many HNPs [9]. In recent years, several studies have shown that HNPs play an essential role in the pathogenesis of thrombotic diseases. The related mechanism may be related to the deposition of HNP1–3 in atherosclerotic human coronary and carotid arteries, impairing lipoprotein metabolism [29] or promoting platelet activation [30–31] and inhibiting tissue plasminogen activator (tPA) -mediated fibrinolysis in vitro [32]. In this study, we found that elevated levels of anti-β_2_GPI/β_2_GPI complexes in clinical samples were associated with increased HNPs levels. This observation is consistent with our in vitro findings, where treatment of neutrophils with the anti-β_2_GPI/β_2_GPI complex resulted in an increase in HNPs release in a time- and concentration-dependent manner.

As mediators of inflammation, HNPs are involved in the occurrence and development of many inflammatory diseases. Chen [33] et al. found that neutrophils can be induced to release ATP under inflammatory stimulation, which in turn acts on the P2Y_2_ receptor of neutrophils to induce neutrophil degranulation and participate in inflammatory response. He Renzhong [22] et al. found that the upregulation of P2Y_2_ receptor in mice with simulated bronchial asthma could induce the degranulation of neutrophils and the release of α-defensins. Taken together, P2Y_2_ receptors play an important role in regulating neutrophil degranulation. In this study, our results confirmed that anti-β_2_GPI/β_2_GPⅠ significantly upregulated the relative level of P2Y_2_ receptor mRNA in vitro compared with the control group, while ATP treatment significantly enhanced HNP1–3 mRNA and cell supernatant levels. We target the P2Y_2_ receptor with suramin, a purinergic receptor antagonist [34–35], suramin treatment decreased HNP1–3 mRNA and cell supernatant levels, indicating that anti-β_2_GPI/β_2_GPⅠ regulated HNP1–3 release through neutrophil P2Y_2_ receptor.

Mitogen-activated protein kinases (MAPKs) are a group of serine/threonine protein kinases. In mammalian cells, MAPKs play an important role in cell proliferation, stress response, apoptosis, immune defense, and other processes [36]. There are three classical MAPK pathways: ERK pathway, JUN N-terminal kinase (JNK) pathway, and P38 pathway [37]. Hao [38] et al. found that p38MAPK, ERK, and phosphatidyl inositol 3-kinase (PI3K) signaling pathways were involved in the activation of neutrophils by anti-neutrophil cytoplasmic antibody (ANCA) induced by complement c5a. With further research, van der Veen [39] et al. also showed that inhibition of P38MAPK pathway activation could eliminate ANCA-induced respiratory burst and degranulation of neutrophils. In our study, we also found that p38MAPK and ERK1/2 pathways were also involved in the activation of neutrophils by anti-β_2_GPI/β_2_GPⅠ, and the phosphorylation of p38MAPK and ERK1/2 was necessary for the increased release of HNP1–3 induced by anti-β_2_GPI/β_2_GPⅠ. Treatment of neutrophils with the P2Y_2_ receptor inhibitor suramin significantly abolished the phosphorylation of p38MAPK and ERK1/2 pathways in neutrophils induced by anti-β_2_GPI/β_2_GPⅠ. This suggests that anti-β_2_GPI/β_2_GPⅠ promotes the release of HNP1–3 through phosphorylation of P38MAPK and ERK1/2 signaling pathways downstream of the P2Y_2_ receptor.

vWF and P-selectin are important markers released during endothelial cell activation [40], vWF can induce leukocyte aggregation, platelet adhesion, and persistent disturbances in blood circulation at sites of activation, thereby promoting blood coagulation. P-selectin primarily exists as a transmembrane protein at activated sites, facilitating leukocyte adhesion. Under specific conditions, P-selectin is also present in a soluble form in plasma, where it contributes to thrombus formation. Therefore, both vWF and P-selectin are critical procoagulant factors released by endothelial cells [41–42]. Previous studies have found that high levels of HNPs in vitro can induce endothelial cells to activate and release active substances [43]. To further validate the mechanism of HNPs involved in thrombosis, we explored the relationship between HNP-1 and endothelial cells. Notably, our study revealed that HNP-1 not only inhibits endothelial cell proliferation but also promotes the increased intracellular expression of vWF and P-selectin in HUVEC. Moreover, it significantly enhances the release of vWF from HUVEC. This effect is mediated through the regulation of the NF-κB signaling pathway in HUVEC. In summary, our study demonstrates that the anti-β_2_GPI/β_2_GPI complex promotes the release of HNPs and thrombosis by activating the P2Y_2_/MAPKs pathway. Specifically, HNPs induce an increase in vWF and P-selectin production in HUVEC, thereby contributing to thrombus formation.

In conclusion, our findings provide new insights into the activation of neutrophils by the anti-β_2_GPI/β_2_GPI complex in thrombosis, suggesting that targeting the activation of neutrophil P2Y_2_ receptors or their downstream pathways may offer novel therapeutic strategies to control thrombotic events induced by the anti-β_2_GPI/β_2_GPI complex. However, it is important to acknowledge the limitations of in vitro data due to potential differences between in vitro conditions and the in vivo human clinical environment. Therefore, investigating the specific effects of HNPs on endothelial cells in vivo will be a critical focus of our future research.

## Supporting information

S1 File Original membrane blotting by Western blotting method. (PDF)

S1 Table The minimum dataset of the experiment. (XLSX)
