# Supplementary material for: Anti-β2GPI/β2GPI complex promotes thrombosis by activating the P2Y2/MAPKs pathway to increase human neutrophil peptides
Source: PLoS One. 2025 May 22;20(5):e0322447. doi: 10.1371/journal.pone.0322447 (PMC12097582; doi:10.1371/journal.pone.0322447)

Figure 5A-p-p38 1

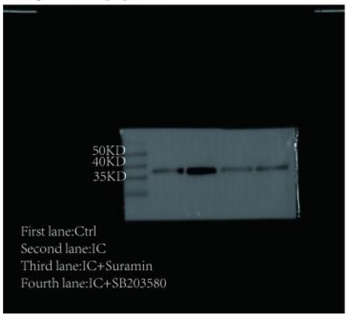

Figure 5A-p-p38 ACTIN 1

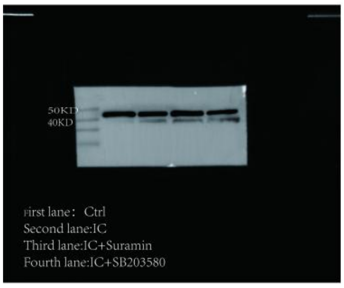

Figure 5A -P-P38 2

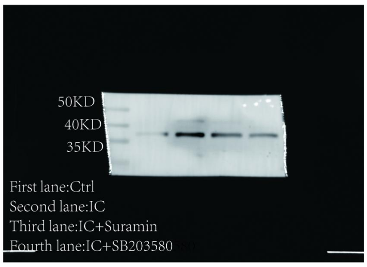

Figure 5A -P-P38 ACTIN2

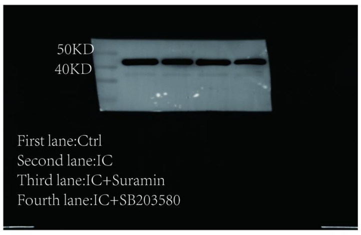

Figure 5A -P-P38 3

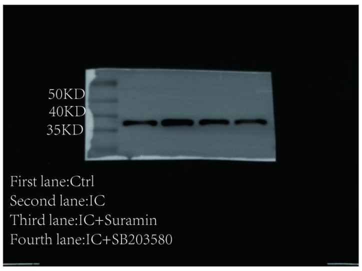

Figure 5A -P-P38 ACTIN3

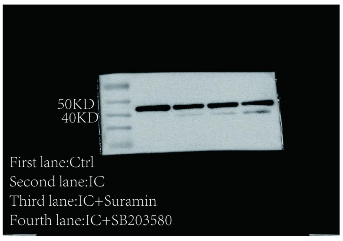

Figure 5B-P-ERK 1

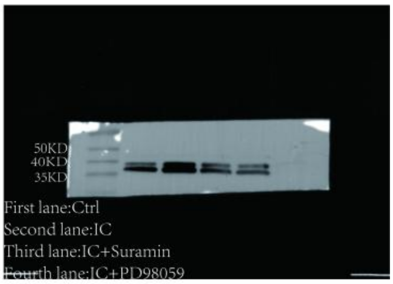

Figure 5B-P-ERK ACTIN 1

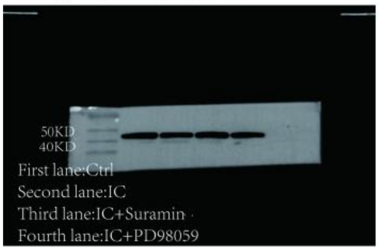

Figure 5B -P-ERK 2

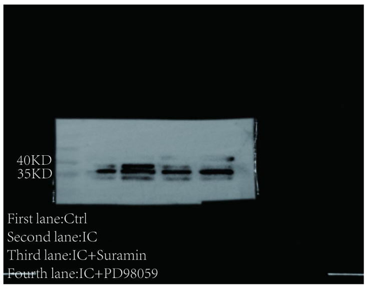

Figure 5B -P-ERK ACTIN 2

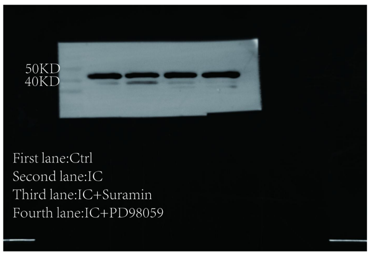

Figure 5B -P-ERK 3

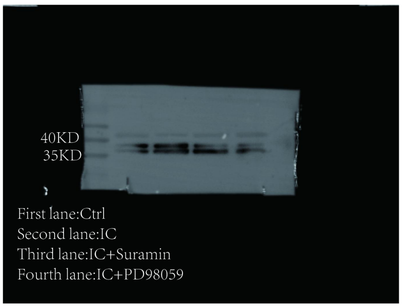

Figure 5B -P-ERK ACTIN 3

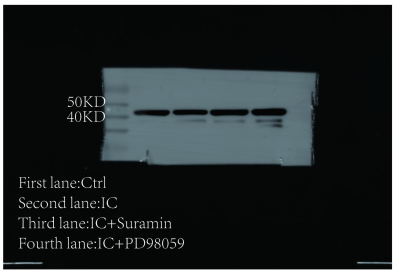

Figure 7A NF-KB P65 1

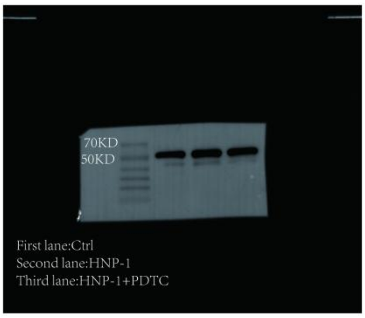

Figure 7A NF-KB P65 ACTIN 1

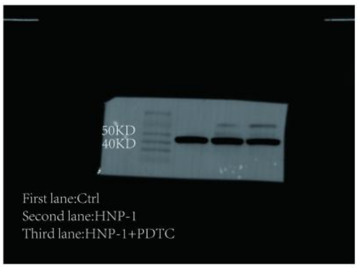

Figure 7A NF-KB P65 2

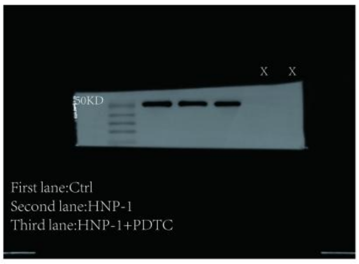

Figure 7A NF-KB P65 ACTIN 2

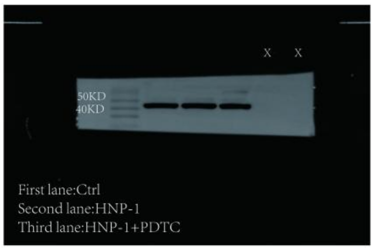

Figure 7A NF-KB P65 3

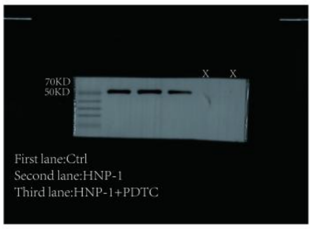

Figure 7A NF-KB P65 ACTIN 3

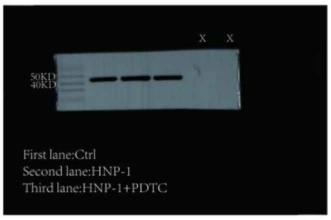

Figure 7A NF-KB P-P65 1

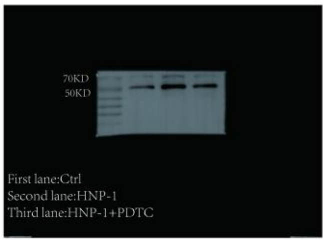

Figure 7A NF-KB P-P65 ACTIN 1

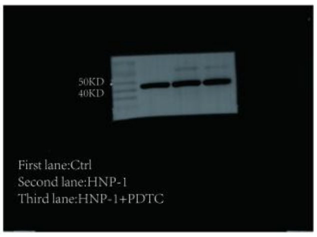

Figure 7A NF-KB P-P65 2

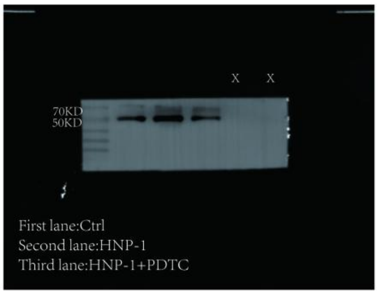

Figure 7A NF-KB P-P65 ACTIN 2

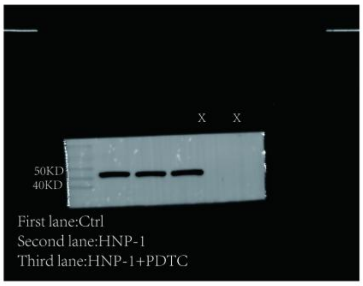

Figure 7A NF-KB P-P65 3

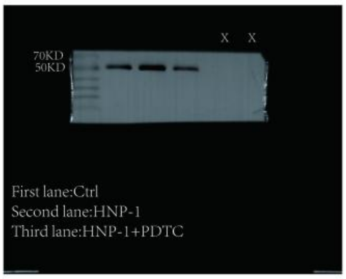

Figure 7A NF-KB P-P65 ACTIN 3

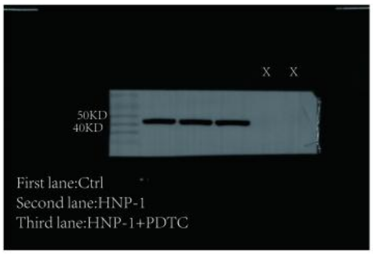

Supplement: S1 File — (PDF) [file pone.0322447.s001.pdf]
